# Changes in Heart Rate Variability after Coronary Artery Bypass Grafting and Clinical Importance of These Findings

**DOI:** 10.1155/2015/680515

**Published:** 2015-05-20

**Authors:** Nenad Lakusic, Darija Mahovic, Peter Kruzliak, Jasna Cerkez Habek, Miroslav Novak, Dusko Cerovec

**Affiliations:** ^1^Department of Cardiology, Krapinske Toplice Hospital for Medical Rehabilitation, School of Medicine Osijek, Gajeva 2, 49217 Krapinske Toplice, Croatia; ^2^Department of Neurology, Zagreb University Hospital Center, School of Medicine, Zagreb, Croatia; ^3^Department of Cardiovascular Diseases, International Clinical Research Center, St. Ann's Faculty Hospital and Masaryk University, Pekarska 53, 656 91 Brno, Czech Republic; ^4^Department of Cardiology, Sveti Duh University Hospital, Zagreb, Croatia

## Abstract

Heart rate variability is a physiological feature indicating the influence of the autonomic nervous system on the heart rate. Association of the reduced heart rate variability due to myocardial infarction and the increased postinfarction mortality was first described more than thirty years ago. Many studies have unequivocally demonstrated that coronary artery bypass grafting surgery generally leads to significant reduction in heart rate variability, which is even more pronounced than after myocardial infarction. Pathophysiologically, however, the mechanisms of heart rate variability reduction associated with acute myocardial infarction and coronary artery bypass grafting are different. Generally, heart rate variability gradually recovers to the preoperative values within six months of the procedure. Unlike the reduced heart rate variability in patients having sustained myocardial infarction, a finding of reduced heart rate variability after coronary artery bypass surgery is not considered relevant in predicting mortality. Current knowledge about changes in heart rate variability in coronary patients and clinical relevance of such a finding in patients undergoing coronary artery bypass grafting are presented.

## 1. Introduction

Sinus rate is neither constant nor uniform but is changing all the time under the influence of the sympathetic and parasympathetic systems. The impact of the autonomic nervous system on the occurrence and mortality of malignant arrhythmias was demonstrated on an experimental animal model as early as some thirty years ago. Decreased parasympathetic tone or increased sympathetic tone predisposes patients to the occurrence of malignant arrhythmias, even ventricular fibrillation. And* vice versa*, increased parasympathetic tone or decreased sympathetic tone reduces myocardial vulnerability and thus the occurrence of ventricular rhythm disturbances [1]. Such unambiguous experimental evidence has encouraged researchers to search for and develop a method to quantitatively measure autonomic nervous activity. Analysis of heart rate variability (HRV) is one of such indicators of the autonomic nervous system activity.

## 2. Heart Rate Variability: Basic Concept and Clinical Use

Heart rate variability is a physiological feature that indicates the effect of the autonomic nervous system on the heart action, that is, heart rate [2]. In 1996, the Task Force of the European Society of Cardiology and the North American Society of Pacing and Electrophysiology issued guidelines on HRV standards of measurement, physiological interpretation, and clinical use [3]. HRV implies two types of changes, that is, variability in the duration of consecutive R-R intervals of the respiratory sinus arrhythmia type and variable heart rate such as sinus tachycardia oscillations on physical exertion, normal diurnal sinus rhythm, and nocturnal sinus bradycardia [3]. HRV is determined by commercial software from electrocardiograms (ECG) of variable duration, mostly 24-hour Holter ECG recording.

The measures used to express HRV have been obtained by analysis of the length of RR interval in the time domain and frequency domain. Only “normal,” nonectopic impulses, that is, those produced by sinus node depolarization, are included in the HRV analysis. In daily clinical routine, standard deviation of all normal RR intervals (SDNN) and mean of R-R intervals for normal beats (Mean RR) are used for HRV measurement and basic analysis.

**Figure 1:**
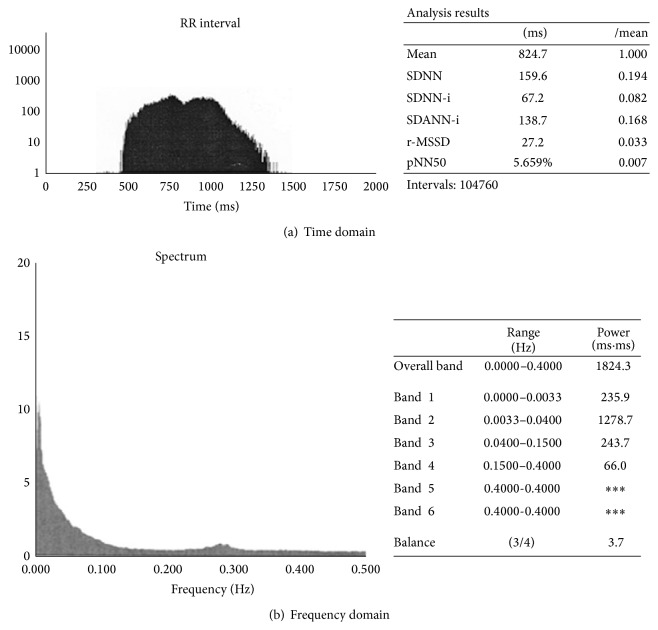
Normal heart rate variability and sympathovagal balance in healthy person (time and frequency domain).

Other HRV measures used in time domain are standard deviation of the 5-minute means of R-R intervals (SDANNi); mean of the 5-minute standard deviations of RR intervals (SDNNi); square root of the mean of the squared successive differences in R-R intervals (rMSSD); and percentage of R-R intervals that are at least 50 ms different from the previous interval (pNN50). The following measures are used in frequency domain: Total Power (range of frequency 0.0–0.5 Hz)—variance of all RR intervals obtained by spectral analysis that corresponds to the SDNN variable in time domain; components of the ultralow frequency spectrum (ULF; 0.0–0.0033 Hz); very low frequency spectrum (VLF; 0.0033–0.04 Hz); low frequency spectrum (LF; 0.04–0.15 Hz); high frequency spectrum (HF; 0.15–0.4 Hz); and their ratio (LF/HF) (Figure 1), [3].

**Figure 2:**
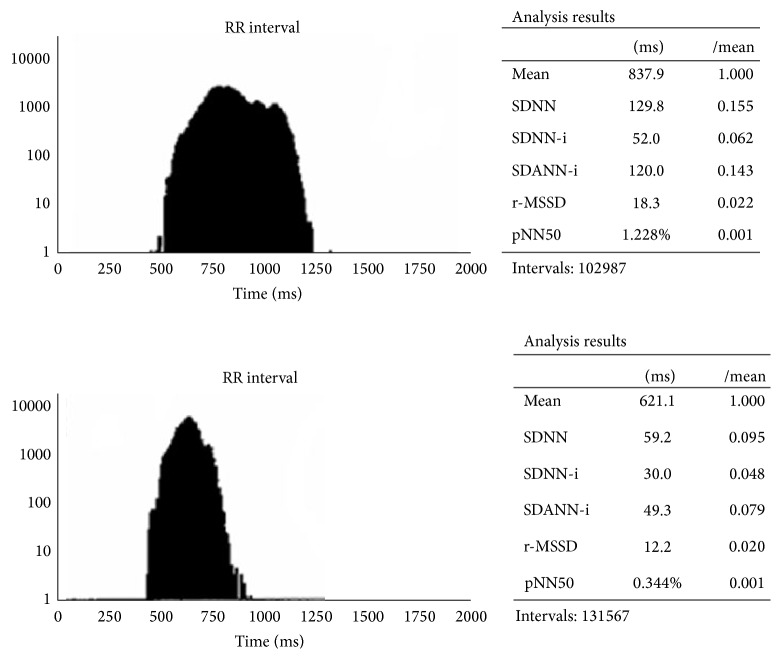
Normal and significantly decreased heart rate variability (HRV) (time domain analysis); see SDNN and other measures.

**Figure 3:**
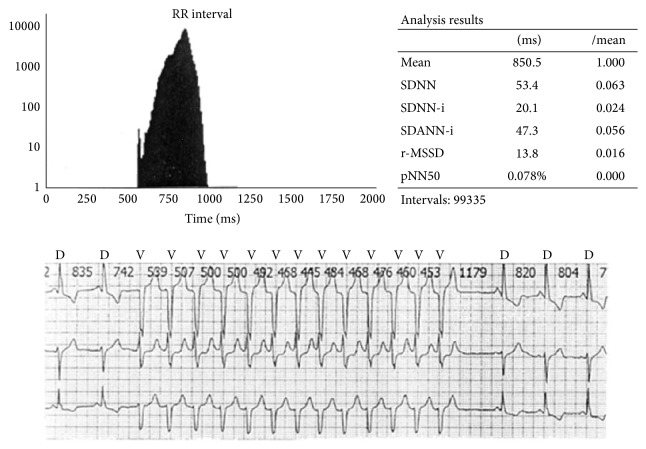
Severely decreased HRV in a patient with subchronic myocardial infarction (see SDNN) and repetitive, nonsustained ventricular tachycardia.

The LF component reflects the sympathetic (and vagal) activity, whereas the HF component along with the rMSSD and pNN50 measures in time domain reflects vagal activity in heart rate modulation. In healthy subjects, the ratio of low frequency and high frequency components (LF/HF) points to the sympathetic and vagal balance, whereas in patients with severely decreased HRV, the LF/HF ratio is very difficult to interpret and its clinical value remains obscure [4]. According to current recommendations [3], SDNN > 100 ms is considered as normal HRV. As the criteria distinguishing pathological from physiological HRV findings have not been clearly identified after release of the guidelines on HRV use [3], Miličević et al. [5] conducted a study on more than 2500 patients in an attempt to define the physiological, moderately decreased, and pathologically decreased HRV values in various groups of cardiac patients. The SDNN < 59 ms was identified as borderline of pathologically decreased HRV and 93 ms as borderline normal HRV, whereas SDNN values of 59–92 ms were found to indicate mildly to moderately decreased HRV in the “general cardiologic population” [5] (Figure 2).  Figure 3 shows pathologically decreased HRV in a patient with subchronic myocardial infarction of the anterior wall and repetitive, nonsustained ventricular tachycardia.

In addition to the above, the researchers also used nonlinear analysis and indices of HRV [6].

## 3. Heart Rate Variability and Myocardial Infarction

Wolf et al. were the first to describe the association of HRV reduction and increased postinfarction mortality in 1978. Analyzing 1-minute ECG recording obtained in a patient with acute myocardial infarction immediately upon admission to coronary unit, they concluded that patients with sinus arrhythmia, that is, with more pronounced sinus impulse variability, had a lower mortality rate than patients with less pronounced variability of sinus impulses [7]. Acute myocardial infarction almost as a rule leads to considerable HRV reduction [8]. This is caused by ischaemia and partial myocardial necrosis. Noncontractile and necrotic left ventricular segments are known to enhance sympathetic afferent and efferent activity, which is manifested as HRV reduction and eventually leads to greater myocardial vulnerability and electrical instability, as well as to the risk of malignant arrhythmias. Furthermore, sympathetic excitation weakens or inhibits vagus influence on the sinus node, which also contributes to lesser heart rate oscillations and HRV reduction. Decreased HRV points to a reduced response of the heart as the target organ to neural modulation inputs or to the impact of sinus node oversaturation by the continuously high sympathetic tone [9, 10].

Bigger Jr. et al. found HRV to be significantly lower in patients having sustained myocardial infarction even a year after the acute coronary event as compared to healthy age-matched subjects [11]. Various other conditions such as heart failure, heart transplantation, stroke, multiple sclerosis, and cardiac surgery procedures can also entail HRV reduction [12–16]. In 1987, Kleiger et al. published their pioneer work demonstrating that patients with a history of myocardial infarction and a higher risk of sudden death could be identified by use of HRV. Analyzing mortality in patients included in the follow-up study, the authors found the patients with decreased HRV, that is, with SDNN < 50 ms, to be at 5.3-fold greater relative risk of death as those with SDNN > 100 ms [17]. This study was followed by a number of other studies that unanimously confirmed the results reported by Kleiger et al. and defined reduced HRV as a strong marker of rhythmogenic death [18–22].

## 4. Heart Rate Variability and Coronary Artery Bypass Grafting

Many studies have invariably demonstrated that coronary artery bypass grafting (CABG) generally leads to significant HRV reduction, which is even more pronounced than after myocardial infarction [16, 23–29]. HRV reduction after cardiac surgery is not exclusively related to CABG, as it is also recorded in patients undergoing valve surgery [30]. Unlike myocardial infarction where the main reason for this is ischaemia and myocyte necrosis, the probable reasons for considerable HRV reduction immediately after CABG include a combined effect of surgical manipulation during operative procedure on the heart and adjacent anatomical structures, prolonged anaesthesia, cardioplegia, and extracorporeal circulation.

Analyzing HRV differences between patients operated on off-pump* versus* on-pump, Kalisnik et al. conclude that off-pump CABG is also followed by extensive adrenergic activation that is comparable to on-pump CABG [31]. Our results also suggested that there were no differences in HRV a few months after surgery between patients undergoing off-pump and patients undergoing on-pump CABG [32].

Generally, in most patients, HRV recovery to the values measured before CABG occurs gradually within six months of the operative procedure [16, 23]. There are reports indicating that a finding of reduced HRV after CABG is of no relevance in predicting mortality, unlike reduced HRV in patients having sustained myocardial infarction [33–35]. To put it more precisely, the authors of those studies conclude that, unlike the strong prognostic potential of HRV in postmyocardial infarction patients, HRV finding has no prognostic value in post-CABG patients. It is explained by revascularization of the ischaemic or viable myocardial tissue, which exceeds the significance of decreased HRV and autonomic dysfunction [34]. Also, Stein et al. conclude that excluding CABG and diabetic patients from HRV analysis significantly increases the relationship of reduced HRV and mortality rate [33, 35]. Contrary to the reports where decreased HRV after CABG had no significant prediction of mortality, the results of our study indicated that postoperative HRV decrease influenced mortality rate in patients after CABG [35]. Unlike some previous studies comparing mortality of patients having sustained myocardial infarction and CABG patients with reduced HRV [34], we analyzed mortality in the group of CABG patients with normal* versus* decreased postoperative HRV, which could at least in part explain differences in the results. In our study, one-third of patients had reduced and two-thirds had normal postoperative HRV, measured at a mean of 3.7 months after CABG, with the average 3-year follow-up after HRV analysis. In the follow-up period, 7.8% of adverse coronary events (death from diagnosed new myocardial infarction or sudden death) were recorded and the majority of patients had decreased HRV (*P* = 0.001) [36].

Accordingly, it is logical to ask why HRV reduction definitely is of prognostic value in one group of patients like those with myocardial infarction, whereas in another group of patients like those undergoing CABG such a finding is at least dubious. HRV is decreased to a certain extent in various clinical conditions, but the underlying mechanisms of this reduction are different and that is why the finding of reduced HRV is of different prognostic relevance. In myocardial infarction, HRV reduction is caused by partial myocardial necrosis, in stroke by cerebral parenchymal necrosis, in hyperthyroidism by the effect of elevated thyroid hormone concentrations in the circulation, and in CABG mostly by surgical manipulation and all other instrumentation such as anaesthesia and cardioplegia. For example, treatment of hyperthyroidism results in decreased thyroid hormone concentration in the circulation, reduced heart rate, and consequently HRV normalization [37]. In addition, comorbidities in each individual patient should always be taken in consideration; in CABG patients, these may include diabetes mellitus, heart failure, and previous myocardial infarction. HRV should also be observed in relation to other relevant indicators available, such as left ventricle ejection fraction and patient functional capacity, and only then clinical conclusions can be made. Thus, while a decreased HRV may objectively be a poor prognostic sign in one patient, in another one it will be so to a much lesser extent.

Yet, reduced HRV persisting for months after CABG should raise suspicion in clinicians, in particular if accompanied by a reduced ejection fraction. As ejection fraction correlates well with HRV parameters, prolonged HRV reduction following CABG can also be perceived as a reflection of the level of ejection fraction damage [3, 38].

In conclusion, it is clear that, in the majority of patients, HRV decreases immediately after CABG, with gradual recovery within a few months of the operation. In our opinion, as a guideline for daily clinical practice, it is still unclear whether decreased postoperative HRV several months after CABG has prognostic relevance for the outcome of CABG patients. Correlation between postoperatively decreased HRV and outcome of CABG patients is controversial and additional studies are needed, the more so as the current guidelines on HRV analysis do not answer this question either [3]. It is necessary to conduct studies in a larger sample of patients, in order to acquire additional knowledge and make definitive conclusion on the prognostic value of post-CABG HRV.

According to the results of our previous study [36], we strongly believe that subgroup of patients with decreased HRV a few months after CABG require careful long-term monitoring, diagnostic evaluation, and wide usage of medications with a well-documented favourable effect on HRV and patient clinical outcome [39–42].
